# Acute coronary syndrome management in hemophiliacs: How to maintain balance?: A review

**DOI:** 10.1097/MD.0000000000033298

**Published:** 2023-03-17

**Authors:** Hao Chen, Shaning Yang

**Affiliations:** a Internal Medicine Resident, Department of Cardiology, The First Affiliated Hospital of Yangtze University, Jingzhou, China; b Department of Cardiology, The First Affiliated Hospital of Yangtze University, Jingzhou, China.

**Keywords:** acute coronary syndrome, hemophilia, hemophilia A, hemophilia B

## Abstract

To analyze existing literature and understand how to balance the minimization of bleeding risk and ensuring adequate anticoagulation during coronary intervention treatment and long-term postoperative anticoagulation in hemophilia patients during the perioperative period, in order to provide guidance for healthcare professionals in developing effective treatment plans. This narrative review will analyze existing studies, case reports, and clinical guidelines to determine the most effective strategies for managing acute coronary syndrome in hemophilia patients. When evaluating the literature, factors such as patient age, medical history, and severity of the condition will be considered. The current management guidelines for acute coronary syndrome in hemophilia patients are not based on systematic evaluation and mainly rely on expert opinions. This article provides a comprehensive analysis of existing literature and recommends coagulation factor replacement therapy before anticoagulation and intervention treatment, as well as personalized anticoagulation treatment during the postoperative period for better management of hemophilia patients with acute coronary syndrome. These recommendations can help healthcare professionals develop more effective treatment plans for hemophilia patients.

## 1. Introduction

Hemophilia is an X-chromosome linked recessive hereditary bleeding disorder, which can be classified into 2 types: hemophilia A and hemophilia B.^[1]^ The former is coagulation factor VIII deficiency, and the latter is coagulation factor IX (FIX) deficiency, caused by mutations in the corresponding coagulation factor genes, respectively.^[1]^ The severity of hemophilia depends on the level of clotting factors. In hemophilia A/B, factor values below1% are considered severe; 1% to 5% are considered moderate and > 5% moderate. Since the advent of clotting factor replacement therapy, the life expectancy of hemophiliacs has been equal to that of the general population.^[2–4]^ However, this also brings a series of new challenges. Increasingly, patients with hemophilia are developing serious complications such as cardiovascular disease, primarily acute coronary syndrome (ACS).^[5]^ Although previous reports suggest that low levels of clotting factors can provide some protection against cardiovascular events,^[6,7]^studies on vascular markers of cardiovascular diseases, including intima-medium thickness of the arteries and coronary artery calcification measurements, now indicate that atherosclerosis develops similarly over time in patients with hemophilia and the general population.^[7–9]^In a recent study, Miesbach et al found that approximately 30% of patients older than 60 years with moderate or severe hemophilia were diagnosed with cardiovascular disease.^[10]^However, there is a lack of evidence-based medical support for the optimal management of patients with hemophilia complicated by ACS, and the existing expert consensus on the management of patients with hemophilia primary percutaneous coronary intervention (PCI) in the preoperative period and long-term postoperative antithrombotic is unclear, and these patients are often not optimally treated and managed.^[11,12]^ Based on a review of the literature, there are no current reviews reported on ACS in hemophiliac patients in China; thus, the purpose of this review is to understand how to strike a balance between minimizing the risk of bleeding and ensuring adequate antithrombotic formation in hemophiliac patients during the perioperative and long-term postoperative antithrombotic period.

## 2. Revascularization

Patients with hemophilia are at increased risk for perioperative bleeding, which can be quite challenging for cardiologists at the time of PCI. Since patients with bleeding disorders are usually excluded from ACS clinical trials, there are currently no guidelines for the management of ACS in patients with hemophilia, and the management is more based on case reports and expert consensus. The therapy of hemophilia complicated by cardiovascular disease is currently not well-trained for cardiologists. Hemophilia specialists need to be consulted in the management of these patients.^[13]^ According to ESC guidelines, PCI is the treatment of choice for early revascularization in patients with ACS.^[14]^ Currently, the latest recommendations for hemophiliacs presenting with high-risk ACS and requiring early hemodialysis should be treated with PCI as promptly as the general population(Table 1).^[5,15]^ Coronary artery bypass grafting (CABG) is reserved for patients with complex coronary artery disease as a 3-vessel disease, left main stenosis or stenosis of the proximal left anterior descending artery.^[13]^ However, depending on the severity of the bleeding disorder, the urgency of the procedure, and the availability of coagulation factor concentrates, replacement therapy should be initiated as soon as possible under the supervision of a hemophilia specialist, before or in conjunction with surgery, but always before removal of the sheath tube.^[15]^ In the perioperative period, peak levels of coagulation factors should exceed 80% of normal values.^[15]^

**Table 1:** The main recommendations for the treatment of ACS in patients with hemophilia.

	Recommendation	References
Reperfusion	PCI is the main line of treatment for STEMI and high-risk NSTEMI hemophiliacs, and it should be performed as soon as possible.	^[5,15]^
	CABG is reserved for patients with complex coronary artery disease as a 3-vessel disease, left main stenosis or stenosis of the proximal left anterior descending artery.	^[13]^
Stent	BMS as the first choice.	^[16,17]^
	New generation DES requiring short DAPT may be used.	^[12,18–21]^
Access site	Radial access is the default access site for PCI. Femoral access may be used if the interventional cardiologist does not have experience in radial access.	^[13,22,23]^
Anticoagulant	UFH is the anticoagulant of choice during PCI.	^[14,15]^
Antiplatelet	Dual antiplatelet therapy should be administered for as short a duration as possible. The ideal duration of DAPT is considered to be 1–6 mo, depending on the type of stent.	^[12,15]^
	Clopidogrel is the preferred P2Y_12_ receptor inhibitor as part of DAPT.	^[15,24]^
Replacement therapy	The alternative coagulation factor therapy should achieve a peak level of at least 80% during PCI, and the minimum level should be maintained at 50% within 24 hours after PCI.	^[5,13]^
	FVIII/FIX trough levels should be ≥ 20% during DAPT.	^[16,25–29]^
	FVIII/FIX trough levels should be ≥ 5%–10% during SAPT.	^[16,26]^
PPI	All patients with hemophilia should receive proton pump inhibitors during DAPT in order to decrease the risk of gastrointestinal bleeding.	^[30–32]^

ACS = acute coronary syndrome, BMS = bare metal stent, DAPT = dual antiplatelet therapy, DES = drug-eluting stent, FVIII = factor VIII, FIX = factor IX, NSTEMI = non-ST-segment elevation myocardial infarction, PCI = percutaneous coronary intervention, STEMI = ST-segment elevation myocardial infarction, UFH = unfractionated heparin.

Several institutions are investigating the complications of cardiovascular disease in patients with hemophilia and have developed expert consensus on perioperative management and long-term postoperative antiplatelet therapy strategies.^[12,15,30,33]^According to the literature, patients with hemophilia who experience acute coronary syndrome can be treated with PCI under coagulation factor replacement therapy, with no significant bleeding or cardiovascular events reported at follow up.^[12,18,19,25]^Christian et al^[34]^ reviewed 54 patients with hemophilia A (n = 45, 83%) or B (n = 9, 17%) who underwent coronary angiography with or without PCI. The results showed that peri-interventional factor substitution was performed in the majority of patients (42 of 54, 78%), although not in all cases. Among the 54 patients, 38 (70%) underwent PCI, which involved balloon dilation (n = 5), bare metal (n = 31), or drug-eluting stents (n = 2). For PCI, un fractioned heparin (n = 24), low molecular weight heparin (n = 2), bivalirudin (n = 4), or no peri procedural anticoagulation at all (n = 8) were used. The study found that PCI was successful in all cases. After stenting, the majority of patients (28 of 33; 85%) were treated with dual antiplatelet therapy (DAPT) (median duration 1 month). The study also reported that major peri procedural bleeding episodes occurred in only 3 of the 54 (6%) patients, and bleeding during follow-up occurred in 11 of 54 (20%) patients. Although the study showed that coronary angiography and PCI in patients with hemophilia can be effective and safe when individualized measures are taken to prevent bleeding, for patients with hemophilia, more often conservative drug treatment is adopted. According to a study by Reilley et al^[35]^, conducted in the United States between 1998 and 2011, which analyzed 237 patients with hemophilia A/B who also had ACS, as well as 148,848 ACS patients serving as controls, it was found that patients with hemophilia tended to opt for conservative treatment using medications. For hemophilia A patients, 55.8% of discharges were treated medically, compared to 32.3% of controls. Medical treatment was also preferred among all subtypes of ACS [non-ST-segment elevation myocardial infarction (NSTEMI), ST-segment elevation myocardial infarction (STEMI), unstable angina] for hemophilia A patients compared to controls. Hemophilia A patients had lower rates of PCI or CABG compared to controls, and there was a higher rate of coagulation factor replacement among hemophilia A cases, although this difference was not significant. The in-hospital death rate was similar for hemophilia A cases and controls. For hemophilia B patients, 50.8% of discharges were treated medically, compared to 32.3% of controls. Medical treatment was preferred among discharges for patients with NSTEMI or STEMI compared to controls, but there was no significant difference in treatment pathway between cases and controls for patients with unstable angina. Hemophilia B patients had lower rates of PCI but higher rates of CABG among individuals with STEMI or unstable angina. The rates of bleeding of hemophilia B and controls were similar among patients who underwent PCI treatment. In China, it is estimated that there are 60,000 to 130,000 patients with hemophilia,^[36]^ but only 4 cases have been recorded. Guo Yang et al^[37]^ reported the first case of a hemophiliac patient who died after suffering from STEMI without receiving antithrombotic or PCI therapy. In 2016, 2 cases were reported in which patients with unstable angina underwent PCI and postoperative antithrombotic therapy without significant complications. ^[38,39]^ The 4th patient had NSTEMI and improved with conservative drug treatment.^[40]^To better treat hemophilia patients with cardiovascular disease, more experience needs to be accumulated in China. Thrombolysis may be considered as a treatment option for patients with STEMI and hemophilia without primary PCI, but only after replacement therapy with coagulation factors.^[12]^

People with Hemophilia A and Hemophilia B are less likely to receive invasive treatments (like PCI or CABG) for ACS compared to those without Hemophilia, even though current guidelines recommend immediate catheterization for patients with STEMI.^[15]^ This could affect the cardiovascular outcome of Hemophilia patients, as they may not receive the necessary treatment due to a fear of bleeding risk. Additionally, Hemophilia patients may be more likely to refuse treatment because they want to avoid bleeding. It is important to educate both providers and patients to address this issue. Therefore, based on current case reports and expert consensus, patients with hemophilia and indications for PCI should not be deferred, provided they receive replacement therapy with coagulation factors.

## 3. Access route

Access-related bleeding accounts for 30% to 70% of total bleeding events in patients undergoing PCI.^[41]^ Radial artery access is shown to significantly improve clinical outcomes by reducing access-related bleeding. Two large randomized trials, the RIVAL trial (n = 7021 ACS patients) and the MATRIX trial (n = 8404 ACS patients), showed significantly lower rates of radial access-related bleeding, surgical access point repair, and transfusion compared to femoral access.^[22,23]^Importantly, there was a significant mortality advantage for patients with trans radial access.^[22,42]^Therefore, radial access is recommended as the first choice for patients with hemophilia to reduce bleeding complications, due to its easily compressible puncture site.^[13]^ However, the largest data collection and analysis of hemophiliac patients undergoing coronary angiography and PCI to date have shown that most coronary interventions in hemophiliac patients were performed through femoral access without significant bleeding complications.^[34]^ This suggests that cardiologists may have extensive experience in femoral artery procedures, which could lead to a reduction in bleeding complications. A closure device is recommended to reduce femoral puncture site bleeding after removal of the sheath.^[43]^ While radial access is typically the preferred approach for patients with hemophilia, it should only be performed by skilled specialists trained in accessing the radial artery to ensure the expected benefits of this method can be achieved.

## 4. Selection of stent

As DAPT is required after coronary stent implantation to prevent intracoronary thrombosis, leads to an increased risk of bleeding. Bare metal stent (BMS) have a short endothelialization time and require DAPT for a short period of time (1 month), reducing the risks of bleeding, but with a higher risk of in-stent restenosis.^[44]^ In contrast, drug-eluting stent (DES) require at least 6 months of dual antibodies and increase the risk of bleeding, but have a lower risk of in-stent restenosis.^[44]^ As a result, there is currently controversy regarding the type of stent implanted in patients with hemophilia. This has led some to believe that DES should be avoided in patients with hemophilia, and BMS has been recommended as the first choice.^[16]^ Mannucci et al^[17]^ also recommended that BMS be preferred in patients with hemophilia and that DAPT be discontinued within 1 month. However, in patients with prior restenosis or an elevated risk of restenosis due to diabetes, DES should be considered.^[13]^ It has been reported in several publications that patients with hemophilia given a new generation DES implanted with appropriate replacement therapy for coagulation factors (coagulation factor trough levels ≥ 15% or ≥ 30%) were given DAPT (1–6 months) and had no bleeding or in-stent stenosis events.^[12,18–20]^

In a randomized double-blind controlled trial,^[21]^the aim was to evaluate the efficacy and safety of second-generation DES (polymer-free umirolimus-coated stent) with BMS, in patients at high-risk of bleeding. All patients (1221 in the DES group and 1211 in the BMS group) received DAPT (aspirin + clopidogrel) for 1 month and thereafter were switched to single antiplatelet therapy (aspirin) and followed up. Finally, the incidence of bleeding was found to be similar in both groups, but the use of DES significantly reduced the incidence of cardiogenic death, myocardial infarction, or in-stent thrombosis events compared with BMS. These findings are consistent with was found in 2 other randomized controlled trials, where next-generation DES was superior to BMS in patients at high bleeding risk who could not tolerate long-term DAPT.^[45,46]^ Overall, DES of the second-generation should be the first choice for patients with a high-risk of bleeding.^[44]^In conclusion, in the current era of PCI, with the use of new DES, the refinement of PCI techniques and the introduction of adjuvant drug therapy, the combination of DES with a short-term DAPT regimen similar to BMS to reduce the risk of subsequent repeat revascularization is an effective and safe approach for patients with hemophilia undergoing PCI.^[25]^

## 5. Antiplatelet therapy

**Figure 1.**
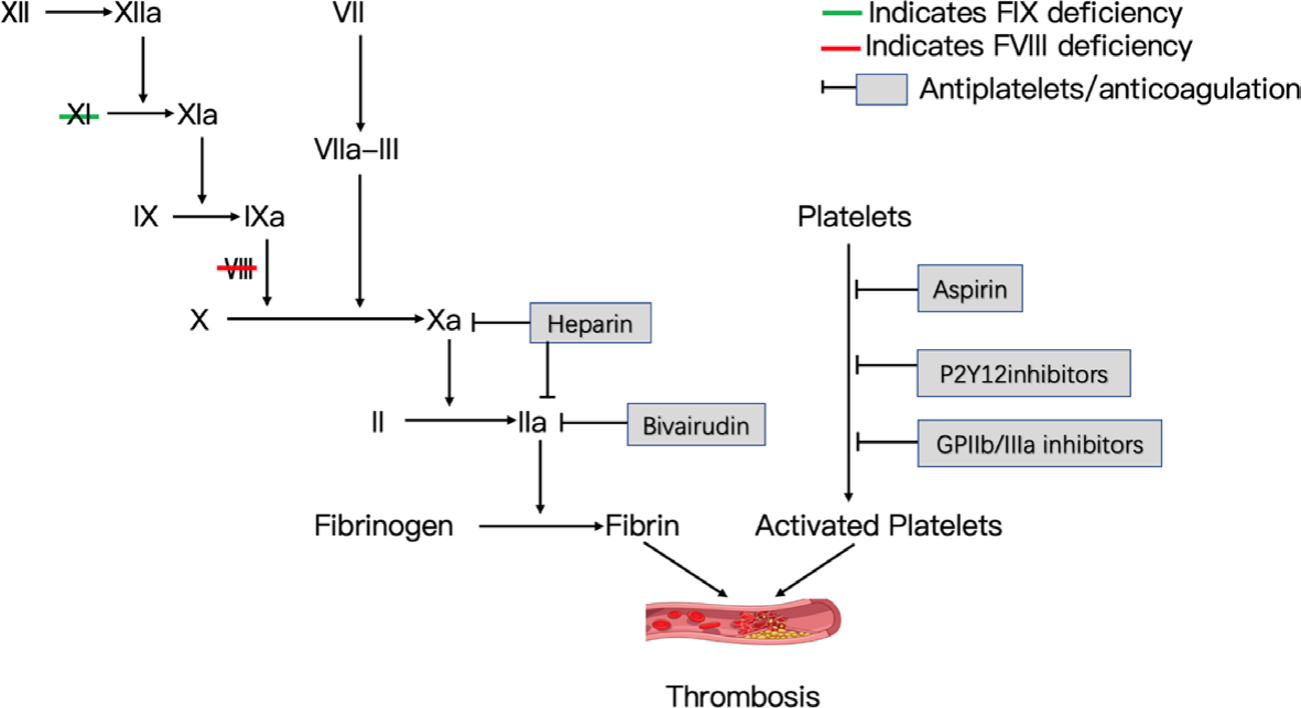
A simplified representation of the coagulation cascade.

DAPT with aspirin and P2Y12 inhibitors (clopidogrel, ticagrelor, prasugrel) (Fig.1)is the cornerstone of percutaneous coronary intervention in patients with ACS.^[44]^ Since platelet function and number are normal in patients with hemophilia, antiplatelet therapy should be given in the setting of ACS.^[15]^ Bovenzi et al reported a case of acute in-stent thrombosis after coronary stenting in a patient with hemophilia B not treated with aspirin or clopidogrel.^[35]^ Regarding the choice of antiplatelet agents, compared to clopidogrel, ticagrelor and prasugrel had a significant reduction in ischemic events after ACS, but the higher efficacy was offset by a higher risk of bleeding.^[24,47]^ A multicenter retrospective cohort study of East Asian patients confirmed that even low doses of prasugrel (loading dose 20 mg, maintenance dose 3.75 mg) were associated with a higher incidence of bleeding events compared with clopidogrel (loading dose 300 mg, maintenance dose 75 mg) (OR, 2.91; 95% CI, 1.63–5.18), and East Asian patients were considered more likely to bleed than Western patients.^[48]^ Overall, clopidogrel is the preferred P2Y12 receptor inhibitor for DAPT in patients with hemophilia.^[15,24]^ Therefore, DAPT consisting of aspirin and clopidogrel is recommended after PCI to prevent in-stent thrombosis.^[15]^The glycoprotein IIb/IIIa (GPIIb/IIIa) receptor antagonists tirofiban and eptifibatide are used as intravenous and intracoronary agents with relatively stable efficacy, acting at the terminal link of platelet aggregation, and are among the potent antiplatelet agents.^[49]^ Because GPIIb/IIIa inhibitors increase the risk of bleeding and have limited efficacy in non-high-risk ACS patients, the ESC recommends their use only in the emergency setting of PCI and in non-ST-segment elevation ACS patients at high ischemic risk undergoing PCI.^[14]^ A case of a patient with moderate hemophilia B with 3 DES implanted for STEMI was recently reported, and intraoperative thrombus load was observed with heavy administration of GPIIb/IIIa inhibitors and postoperative maintenance for 6 hours without significant complications.^[12]^ However, the ADVANCE panel avoided the use of GPIIb/IIIa as much as possible, considering the high-risk of bleeding in patients with hemophilia and the strong antiplatelet effect of GPIIb/IIIa.^[15]^

## 6. Anticoagulant therapy

During revascularization in hemophiliacs, in addition to antiplatelet therapy during PCI, anticoagulation (Fig.1) should be considered after appropriate replacement therapy.^[15]^ In patients with bleeding disorders, the short half-life and easy reversal of anticoagulants become a major consideration. The short half-life and ease of reversal of unfractionated heparin (UFH) and its extensive experience in use make it the anticoagulant of choice in primary PCI.^[14,15]^ Low molecular weight heparin has the advantage of having a lower incidence of bleeding complications^[14]^ and heparin-induced thrombocytopenia^[50]^ compared with UFH, but its effects are more difficult to control and not easily reversed, and therefore not preferred agents. Activated clotting time is prolonged in patients with hemophilia, but coagulation factor replacement therapy can suppress this phenomenon.^[25]^ Activated clotting time remains a useful bedside test to monitor coagulation during PCI and to guide UFH therapy.

Bivalirudin is a direct thrombin inhibitor with a short half-life and a significant reduction in bleeding complications.^[51]^In the MATRIX trial, 7213 ACS patients (56%of STEMI) were enrolled, bivalirudin significantly reduced the risk of bleeding compared to UFH, at the cost of an increased risk of thrombosis in the stent.^[52]^ The ESC guidelines recommend its use in patients with STEMI (IIa, A), especially those at high-risk of bleeding.^[14]^ In patients with hemophilia, bivalirudin has been successfully used in some cases without complications such as bleeding or in-stent thrombosis associated with these drugs.^[53–56]^ However, the specific antidotes for these drugs are not yet approved for use and therefore, the ADVANCE group currently does not recommend bivalirudin in patients with hemophilia, although this may change soon with the development of specific antidotes.^[15,16]^

## 7. Management of bleeding risks associated with anticoagulation and antiplatelet therapy

The incidence of major bleeding events was significantly higher in hemophilia treated with antithrombotic therapy and was directly related to the severity of hemophilia, the presence of prophylaxis and the type of antithrombotic therapy.^[30]^ There is no strong evidence on the optimal level of coagulation factors required during antithrombotic therapy, so expert opinion is essential to guide clinicians in the management of rare conditions, which may increase as the hemophilia population ages. The current consensus is that patients with hemophilia should be given replacement therapy before receiving anticoagulant/antiplatelet drugs. In cases where early PCI (within 90 minutes) is not possible, fibrinolysis may be used, but coagulation factors should be checked immediately to aim for 50% trough levels and 80% peak levels.^[13]^ According to the recommendations of the World Federation of Hemophilia, patients with hemophilia A undergoing major surgery (coronary artery bypass graft) should be supplemented with F VIII prior to the procedure to maintain 80% to 100% peak levels.^[5]^ However, there is no similar protocol for coagulation supplementation prior to PCI. The ADVANCE panel recommends that coagulation factor trough levels should be maintained at approximately 50% for 24 hours after PCI.^[13]^ However, no uniform criteria have been obtained for the optimal level of coagulation factors during antiplatelet. Different expert groups have different opinions, for example: F VIII trough levels, ≥5% to 10%^[16,26]^ during SAPT and ≥ 20% to 30%^[26,30,34]^ or ≥ 30%^[16,25–29]^ during DAPT. Jacob et al^[12]^ reported a case of a patient with mild hemophilia B (baseline FIX level 3–4%) complicated by NSTEMI, who was given DAPT for 6 months after PCI to maintain a FIX trough level of 19% to 23% and subsequently transitioned to SAPT treatment without FIX replacement therapy. Patients were free of any bleeding events and cardiovascular symptoms during treatment. This inconsistency reflects the current lack of evidence-based medical support for the management of patients with hemophilia; therefore, coagulation factor replacement therapy for patients with hemophilia should be based on the severity of the patient’s disease, the type of antithrombotic medication, and, importantly, timely consultation with a hemophilia specialist to guide treatment.

On the other hand, gastrointestinal bleeding is the most common serious complication of long-term antiplatelet therapy.^[31]^ The COCHE study, a prospective, multicenter case-control study (July 2011–December 2017), showed that gastrointestinal bleeding significantly exceeded that of the control group in the antiplatelet therapy group of patients with hemophilia (OR = 15; 95% CI:1.84–268; *P* = .0141).^[30]^ And randomized controlled trials showed that proton pump inhibitors reduced the rate of gastrointestinal bleeding in high-risk patients receiving aspirin.^[32]^ Therefore, once antithrombotic therapy is initiated, PPI should be used for gastroprotection whenever possible.

## 8. Conclusion

Acute coronary syndrome in hemophilia patients is a relatively rare clinical situation that requires close cooperation between experienced cardiologists and hemophilia specialists to maintain a delicate balance between thrombosis prevention and hemorrhage. Early PCI should be administered, starting with radiation access, to patients with hemophilia with high-risk ACS and requiring early revascularization, and it is safe to administer anticoagulation and antiplatelet aggregation therapy under coagulation factor replacement therapy.

## Author contributions

**Supervision:** Shaning Yang.

**Writing – original draft:** Hao Chen.

**Writing – review & editing:** Shaning Yang.
